# Supplementary material for: Extending the defect tolerance of halide perovskite nanocrystals to hot carrier cooling dynamics
Source: Nat Commun. 2024 Sep 16;15:8120. doi: 10.1038/s41467-024-52377-4 (PMC11405528; doi:10.1038/s41467-024-52377-4)
Supplement: Supplementary file 1 — Supplementary Information [file 41467_2024_52377_MOESM1_ESM.pdf]

Extending the Defect Tolerance of Halide Perovskite Nanocrystals to Hot Carrier Cooling Dynamics

Junzhi Ye[1,2#], Navendu Mondal[3#*], Ben P. Carwithen[3], Yunwei Zhang[4], Linjie Dai[1,5], Xiangbin Fan[6], Jian Mao[5,7], Zhiqiang Cui[4], Pratyush Ghosh[1], Clara Otero-Martínez[8], Lars van Turnhout[1], Yi-Teng Huang[2], Zhongzheng Yu[1], Ziming Chen[3], Neil C. Greenham[1], Samuel D. Stranks[1, 5], Lakshminarayana Polavarapu[8], Artem Bakulin[3], Akshay Rao[1], Robert L.Z. Hoye[2,9*]

1. Cavendish Laboratory, University of Cambridge, 11880, Cambridge CB3 0HE, United Kingdom
2. Inorganic Chemistry Laboratory, University of Oxford, South Parks Road, Oxford OX1 3QR, United Kingdom
3. Department of Chemistry and Centre for Processable Electronics, Imperial College London, Molecular Sciences Research Hub, 82 Wood Lane, London W12 0BZ, United Kingdom
4. School of Physics, Sun Yat-sen University, 510275 Guangzhou, China
5. Department of Chemical Engineering and Biotechnology, University of Cambridge, Cambridge CB3 0AS, United Kingdom.
6. Department of Engineering, University of Cambridge, 9 JJ Thomson Avenue, Cambridge, CB3 0FA, United Kingdom
7. State Key Laboratory of Photovoltaic Science and Technology, Shanghai Frontiers Science Research Base of Intelligent Optoelectronics and Perception, Institute of Optoelectronics, Fudan University, Shanghai, 200433, China
8. CINBIO, Universidade de Vigo, Materials Chemistry and Physics Group, Department of Physical Chemistry, Campus Universitario As Lagoas, Marcosende, 36310 Vigo, Spain
9. Department of Materials, Imperial College London, Exhibition Road, London SW7 2AZ, United Kingdom.

* Email: n.mondal@imperial.ac.uk (N.M.), robert.hoye@chem.ox.ac.uk (R. L. Z. H.)

# These authors contribute equally to this work

Supplementary Fig. 1 | Transmission Electron Microscopy S3

Supplementary Fig. 2 | Defect analysis based on compositional analysis of X-ray photoelectron spectroscopy and optical analysis of photothermal defection spectroscopy S4

Supplementary Fig. 3 | Compositional analysis of X-ray photoelectron spectroscopy S4

Supplementary Fig. 4 | Time-correlated Single Photon Counting Measurements S5

Supplementary Fig. 5 | Configuration coordinate diagram describing the interaction between charge-carriers and shallow or deep traps in perovskite NCs S7

Supplementary Fig. 6 | Computational investigation into the effect of defect states in the bulk of $CsPbX_3$ on their electronic structure and density of states S8

Supplementary Fig. 7 | Short-time transient absorption signal decomposition S9

Supplementary Fig. 8 | Hot carrier cooling kinetics, based on pump-probe transient absorption spectroscopy for $CsPbBr_3$ NCs S10

Supplementary Fig. 9 | Hot carrier cooling kinetics based on pump-probe transient absorption spectroscopy for $CsPbBr_xI_{3-x}$ NCs S12

Supplementary Fig. 10 | Hot carrier cooling kinetics based on pump-probe transient absorption spectroscopy for $CsPbI_3$ NCs S14

Supplementary Fig. 11 | Selection of the push energy S16

Supplementary Fig. 12 | Kinetics of GSB and PAs for the representative case of $CsPb(Br/I)_3$ NCs under PP and PPP-TA measurements S16

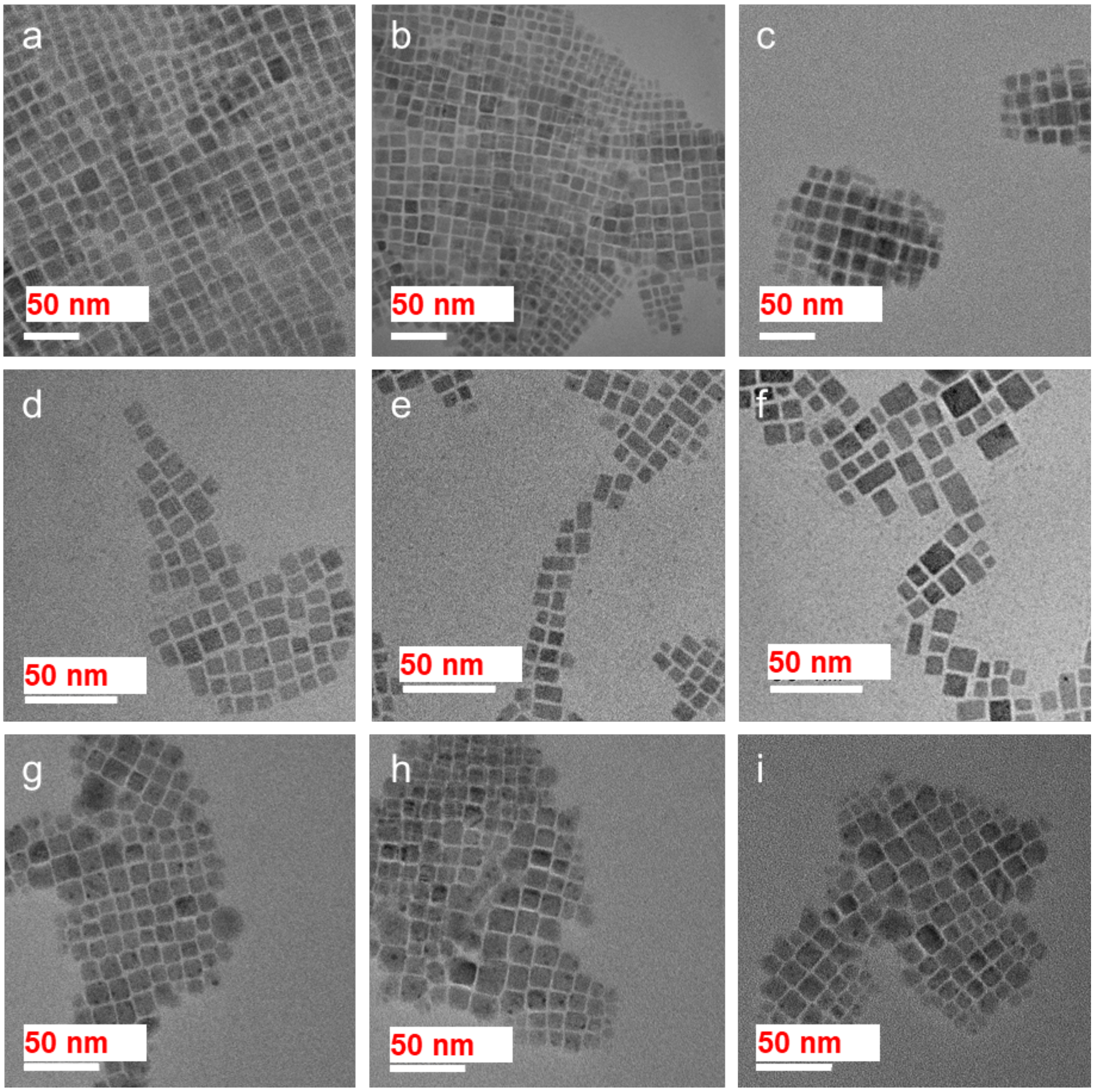

Supplementary Fig. 1 | Transmission Electron Microscopy. a-c, TEM images for pristine (low defect density), single-purified (moderate defect density), and doubly-purified (high defect density) $CsPbBr_3$ perovskite nanocrystal (NCs), respectively. **d-f,** TEM images for pristine (low defect density), single-purified (moderate defect density), and doubly-purified (High Defects) $CsPbBr_xI_{3-x}$ NCs, respectively. **g-i,** TEM images for pristine (low defect density), single-purified (moderate defect density), and doubly-purified (high defect density) $CsPbI_3$ NCs, respectively.

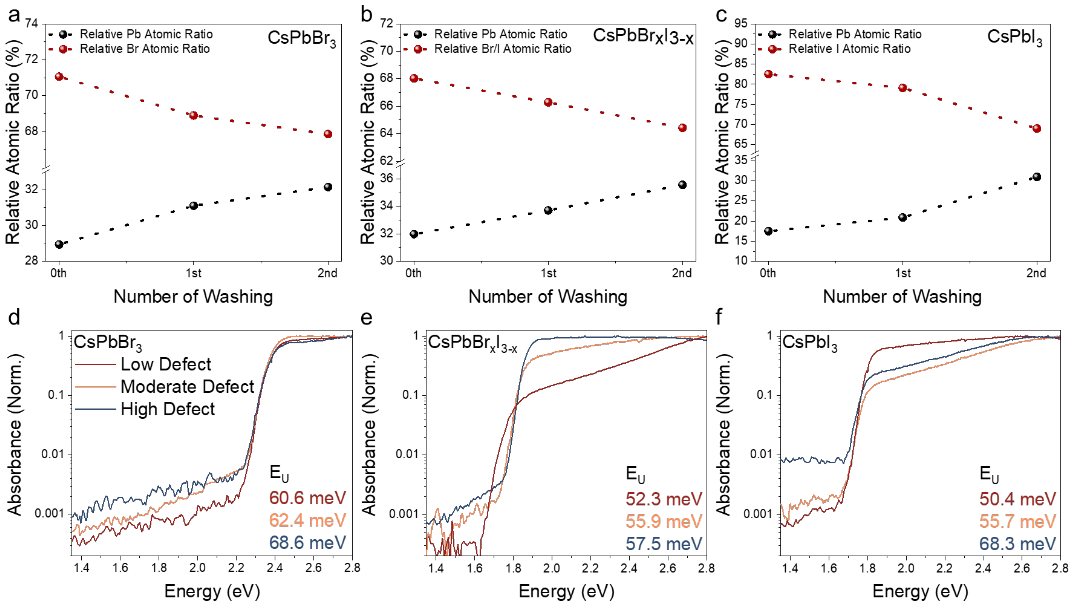

Supplementary Fig. 2 | Defect analysis based on compositional analysis of X-ray photoelectron spectroscopy and optical analysis of photothermal defection spectroscopy. a, Relative Pb/Br ratio for $CsPbBr_3$ NCs after washing. **b.** Relative Pb/Br and I ratio for $CsPbBr_xI_{3-x}$ NCs after washing. **c.** Relative Pb/I ratio for $CsPbI_3$ NCs after washing. **d-e,** Photothermal deflection spectroscopy (PDS) measurement for $CsPbX_3$ NCs with different defect densities and their Urbach energy. The PDS results and Urbach energy fitting is measured and calculated following our previous work[1].

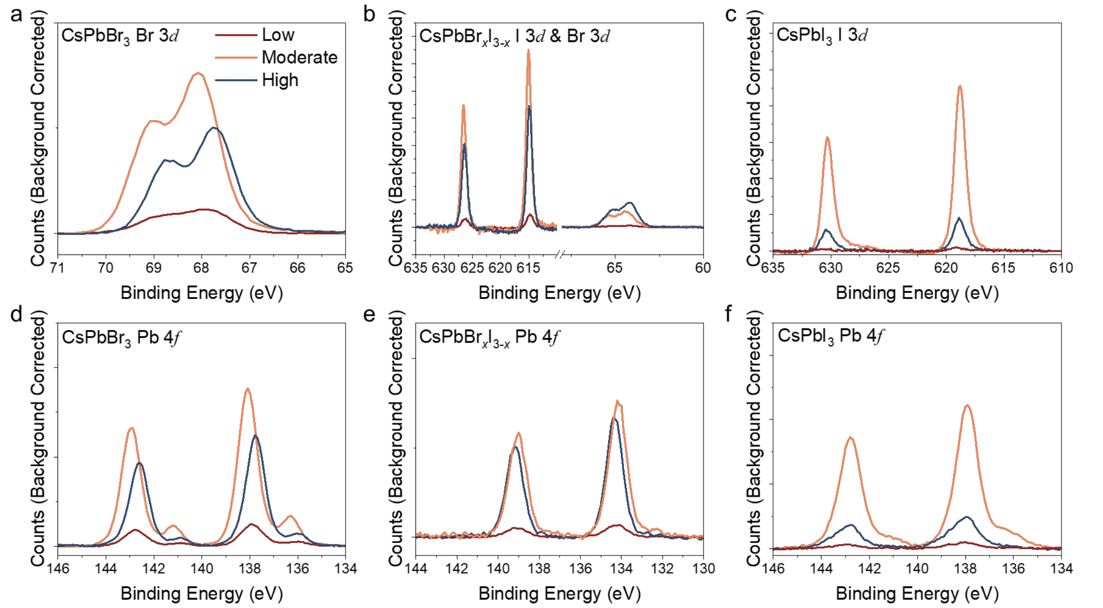

Supplementary Fig. 3 | Compositional analysis of X-ray photoelectron spectroscopy. a-c, Halide (I and Br) 3*d* core level XPS spectra. **d-f,** Pb 4*f* core level XPS spectra of low, moderate and high defect samples. The integrated area ratio between halide and Pb are shown in Supplementary Fig. 2.

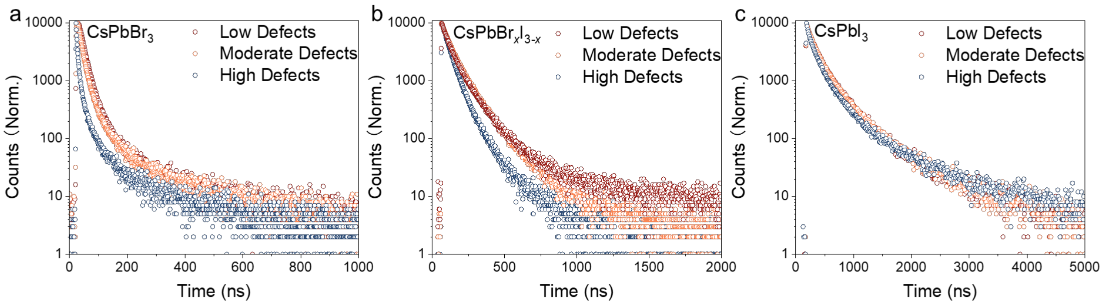

Supplementary Fig. 4 | Time-correlated Single Photon Counting Measurements. Influence of defects densities on photoluminescence lifetime for **a,** $CsPbBr_3$, **b,** $CsPbBr_xI_{3-x}$ and **c,** $CsPbI_3$ NCs. Measured with 405 nm wavelength pulsed laser.

Supplementary Note 1: Hot Carrier Trapping Model

To understand the carrier dynamics in these NCs with respect to the change of pump energy or excess energy above the bandgap, we fit our excitation-dependent PLQY data with a modified rate equation developed by Righetto *et al.*[2] (Supplementary Eq. S1):

$$-\frac{dn}{dt} = k_1 n + k_2 n^2 + k_3 n^3 = k_2 n^2 + \int_0^\infty k_t(\epsilon) n(\epsilon, t) d\epsilon + e^{\frac{\Delta E}{\mathrm{k_B} T}} \int_0^\infty k_t(\epsilon) n(\epsilon, t) d\epsilon \qquad \text{(S1)}$$

where n is carrier density, k_1 is trap-assisted non-radiative recombination rate constant, k_2 is the bimolecular radiative recombination rate constant, and k_3 is the Auger recombination rate constant which is negligible in this case since we measured the PLQY at relatively low excitation power. The modified model implies that the k_1 rate constant is governed by the Marcus theory of charge transfer from free carriers to the potential energy surfaces of trap states, and is correlated the trap transfer rate, k_t with the excess energy that the excited carriers have (δ). We then arrive at Supplementary Eq. S2.

$$k_t(\delta) = \frac{2\pi}{\hbar} |H|^2 \frac{1}{\sqrt{4\pi\lambda \mathrm{k_B} T_\mathrm{L}}} e^{-\frac{(\lambda+\epsilon+\Delta E)^2}{4\lambda \mathrm{k_B} T_\mathrm{L}}} \qquad \text{(S2)}$$

where k_1 and k_2 are related to the trapped carrier rate constant k_t, H is electronic coupling constant to the trap, T_L is the lattice temperature, ϵ is the carrier energy, λ is the reorganization energy and ΔE is the energy offset between trap state and band-edge. The energy diagram is shown in Supplementary Fig. S5. We also assumed that

the carrier distribution (f) is governed by the Boltzmann distribution and it is related to the overall carrier densities, which is shown in Supplementary Eq. S3:

$$n = \int_0^{\infty} g(\epsilon) f(\epsilon) = e^{-\frac{\epsilon-\mu}{\mathrm{k_B}T}} \qquad \text{(S3)}$$

where $g(\epsilon)$ is the assumed parabolic band of the trap states and μ is the chemical potential and assumed to be constant, T is the carrier temperature which the cooling profile follows an exponential decay over time, the temperature profile is governed by Supplementary Eq. S4:

$$T(t) = 300 + \frac{\delta_{\mathrm{E}}}{\mathrm{k_B}} e^{-\frac{t}{\tau_{\mathrm{c}}}} \qquad \text{(S4)}$$

Where δ_E is the excess energy above the bandgap and τ_{c} is cooling time constant. Then we assumed that our PLQY (assuming the radiative recombination is from bimolecular free carrier recombination, since the NCs are in weakly confined regime) can be estimated based on the following equation with respect to the excess energy δ_E:

$$PLQY = \frac{k_2 \int_0^{\infty} n(t)^2 dt}{n_0} \qquad \text{(S5)}$$

Supplementary Table 1 | Fitted parameters for the trapping model in Supplementary Eq. S1–S5 fit to the excitation-energy-dependent PLQY

	$CsPbBr_3$		$CsPbBr_xI_{3-x}$		$CsPbI_3$	
Defects	High	Low	High	Low	High	Low
k_2 (10^{-9}) [a]	0.83±0.00	0.98±0.01	0.87±0.02	0.97±0.01	0.95±0.01	0.95±0.01
Constant [b]	0.09±0.00	0.03±0.01	0.09±0.01	0.04±0.01	0.11±0.01	0.09±0.01

[a] The unit is $cm^3\ s^{-1}$

[b] The fitting constant is related to the trap transfer constant (k_t) which determined by ΔE (the energy offset between trap state and bandedge) and H (electronic coupling constant to the trap); please refer to Supplementary Eq. S2. The fitted values may not be absolutely accurate, but the qualitative changes in these values are sensible. That is, higher defect-density perovskite nanocrystals have stronger electronic coupling to traps and lower radiative recombination constants (k_2).

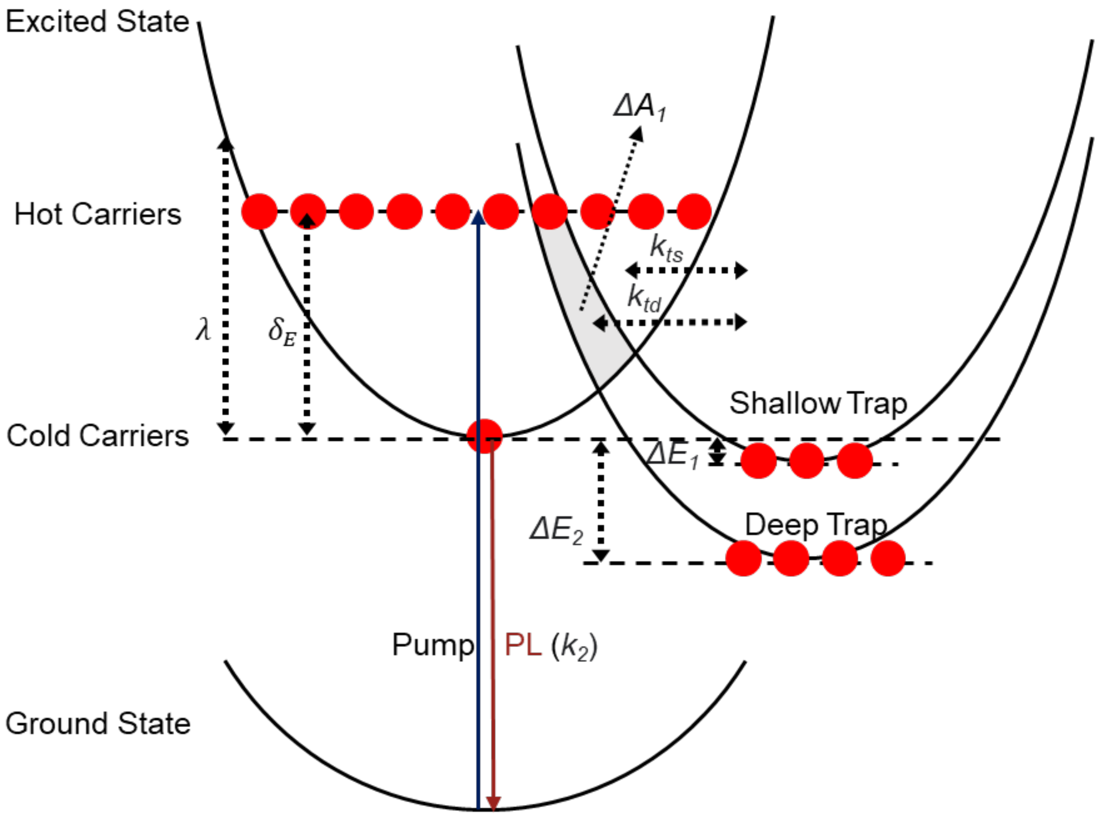

Supplementary Fig. 5 | Configuration coordinate diagram describing the interaction between charge-carriers and shallow or deep traps in perovskite NCs.

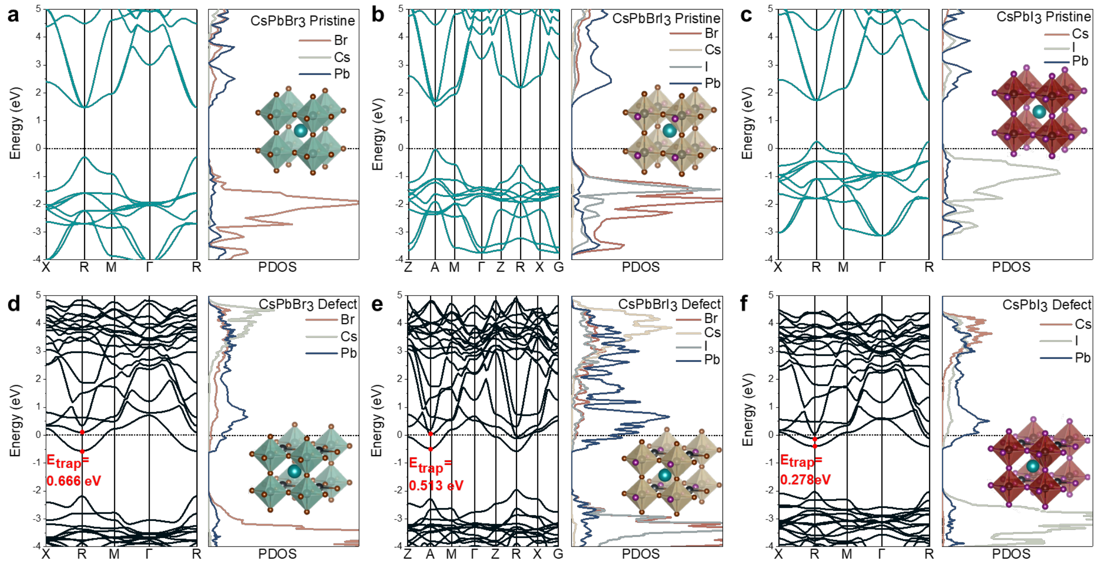

Supplementary Fig. 6 | Computational investigation into the effect of defect states in the bulk of $CsPbX_3$ on their electronic structure and density of states. Band structures and projected density of states of pristine (no defects) **a,** $CsPbBr_3$, **b,** $CsPbBr_xI_{3-x}$, and **c,** $CsPbI_3$, along with defective **d,** $CsPbBr_3$, **e,** $CsPbBr_xI_{3-x}$, and **f,** $CsPbI_3$. The defective materials were simulated as having two halide vacancies per unit cells. Please note that this simulation was performed on bulk materials, not NCs, and does not account for surface defects. Details of how these calculations were performed are provided in the Methods section.

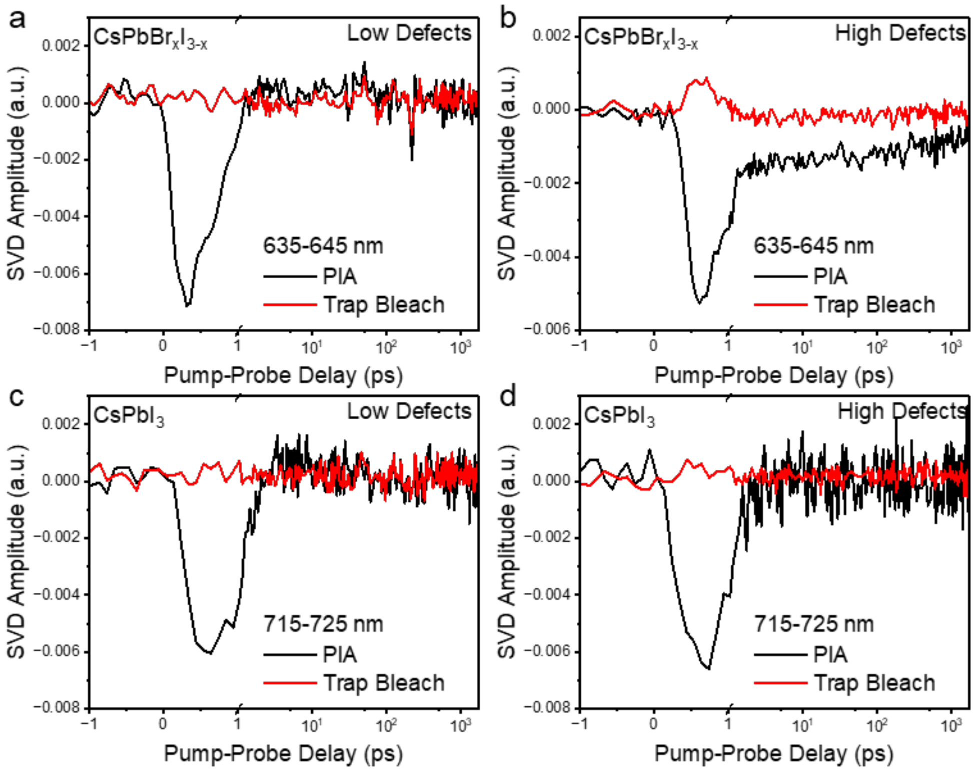

Supplementary Fig. 7 | Short-time transient absorption signal decomposition. Spectra deconvolution for **a,** low defects $CsPbBr_xI_{3-x}$ NCs and **b,** high defects $CsPbBr_xI_{3-x}$ NCs. Spectra deconvolution for **c,** low defects $CsPbI_3$ NCs and **d,** high defects $CsPbI_3$ NCs. The decomposition method is used in our previous report[3].

The decay shown in Fig. 2 d-e of the main text can be decomposed into two components: a positive bleach component (red curve) due to trap filling, and a negative photoinduced absorption (PIA; black curve). The NCs with high defect densities in Supplementary Fig. 7b shows a clear positive trap bleach decay compared to the NCs with low defect densities. The TB for the mixed I/Br-based perovskites are less obvious than the Br-based NCs as shown in main text Fig. 2c, as the trap positions are shallower than Br-based NCs, leading to greater defect tolerance. When moving to even shallower I-based NCs, there is no obvious positive trap bleach signal regardless the level of defects in the systems, indicating less hot carrier trapping process in the sub-bandgap position.

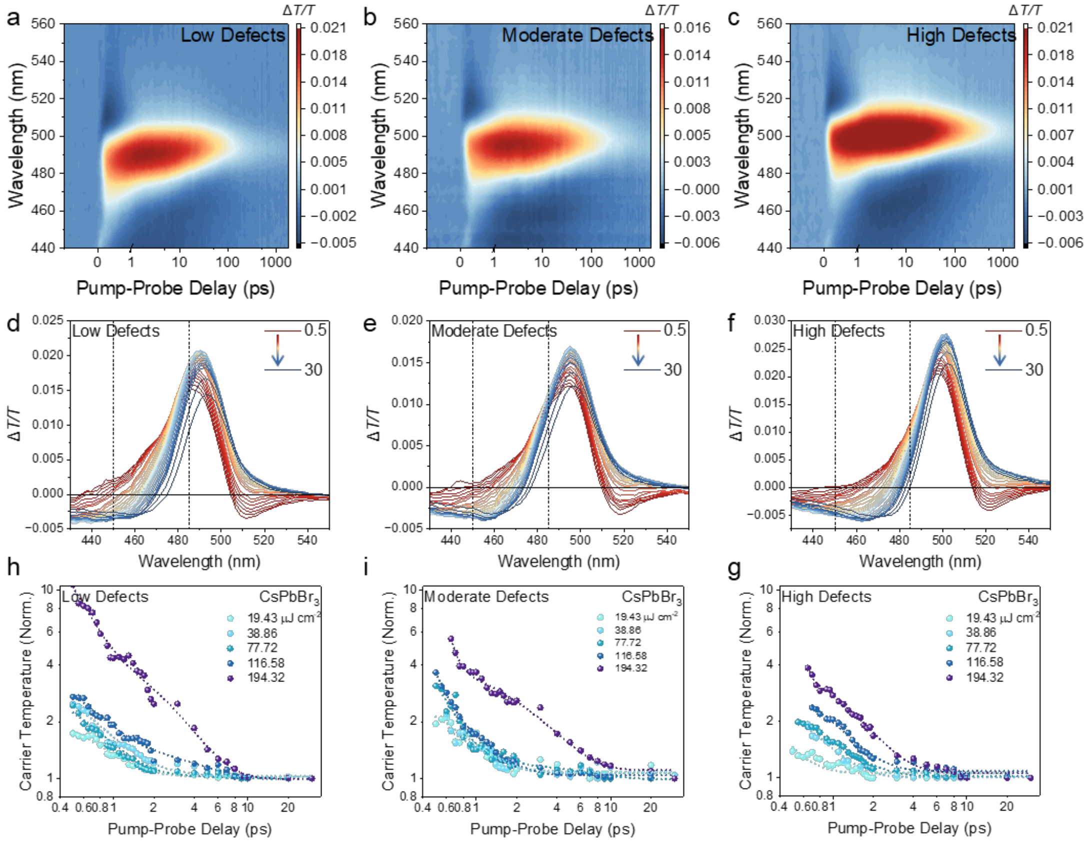

Supplementary Fig. 8 | Hot carrier cooling kinetics, based on pump-probe transient absorption spectroscopy for $CsPbBr_3$ NCs. a-c, TA maps, **d-f,** TA spectra and **h-g,** extracted charge-carrier temperatures *vs*. pump-probe delay of **a, d, h,** pristine (low defect density), **b, e, i,** single-purified (moderate defect density), and **c, f, g,** doubly-purified (high defect density) $CsPbBr_3$ perovskite nanocrystal solutions. The pump wavelength was 400 nm, repetition rate 500 Hz, and the maps and spectra were recorded under a fluence of 194.32 µJ cm^{-2}. In **d-f**, the fitting window for obtaining hot carrier cooling kinetics is indicated with the dashed lines, and was chosen to cover the relevant energy range for hot carriers, whilst not being affected by the decay in the GSB.

Supplementary Table 2 | Fitted Cooling Kinetics for $CsPbBr_3$ NCs shown in Fig. 4a. The number of decimal places quoted for the mean values and uncertainties were obtained based on numerical fitting of the carrier temperature vs pump-probe delay time. We rounded to 1 d.p. to not imply that we can measure with better than 100 fs accuracy. A biexponential model was applied to fit the decay in the carrier temperature over time. At low fluence, the overall time constants ($t_{average}$) mostly follow t_1, indicating a monoexponential decay.

Fluence μJ cm^{-2}	Carrier Density cm^{-3}	$t_{average}$ (ps)	t_1 (ps)	A_1	t_2 (ps)	A_2
Pristine (low defect density)						
19.43	1.25×10^{17}	0.5±0.1	0.5±0.1	100.0%	-	-
38.86	2.50×10^{17}	0.6±0.1	0.5±0.1	96.8%	1.8±0.1	3.2%
77.72	5.00×10^{17}	1.5±0.1	0.6±0.1	94.3%	3.9±0.1	5.7%
116.58	7.50×10^{17}	3.4±0.2	0.6±0.2	65.5%	4.2±0.3	34.5%
194.32	12.49×10^{17}	3.8±0.3	0.7±0.2	61.3%	4.6±0.4	38.7%
Singly-purified (moderate defect density)						
Fluence μJ cm^{-2}	Carrier Density cm^{-3}	$t_{average}$ (ps)	t_1 (ps)	A_1	t_2 (ps)	A_2
19.43	1.25×10^{17}	0.3±0.1	0.3±0.1	100.0%	-	-
38.86	2.50×10^{17}	0.5±0.1	0.3±0.0	88.1%	1.0±0.1	11.9%
77.72	5.00×10^{17}	0.9±0.2	0.4±0.1	84.6%	1.5±0.3	15.4%
116.58	7.50×10^{17}	1.4±0.3	0.5±0.3	71.3%	2.0±0.3	28.7%
194.32	12.49×10^{17}	2.6±0.4	0.6±0.1	69.7%	3.5±0.5	30.3%
Doubly-purified (high defect density)						
Fluence μJ cm^{-2}	Carrier Density cm^{-3}	$t_{average}$ (ps)	t_1 (ps)	A_1	t_2 (ps)	A_2
19.43	1.25×10^{17}	0.3±0.2	0.3±0.2	100.0%	-	-
38.86	2.50×10^{17}	0.1±0.1	0.3±0.0	65.0%	0.5±0.1	35.0%
77.72	5.00×10^{17}	0.6±0.1	0.4±0.0	60.6%	0.7±0.1	39.4%
116.58	7.50×10^{17}	0.6±0.1	0.5±0.1	56.7%	0.8±0.1	43.3%
194.32	12.49×10^{17}	1.0±0.1	0.5±0.0	45.5%	1.1±0.1	54.5%

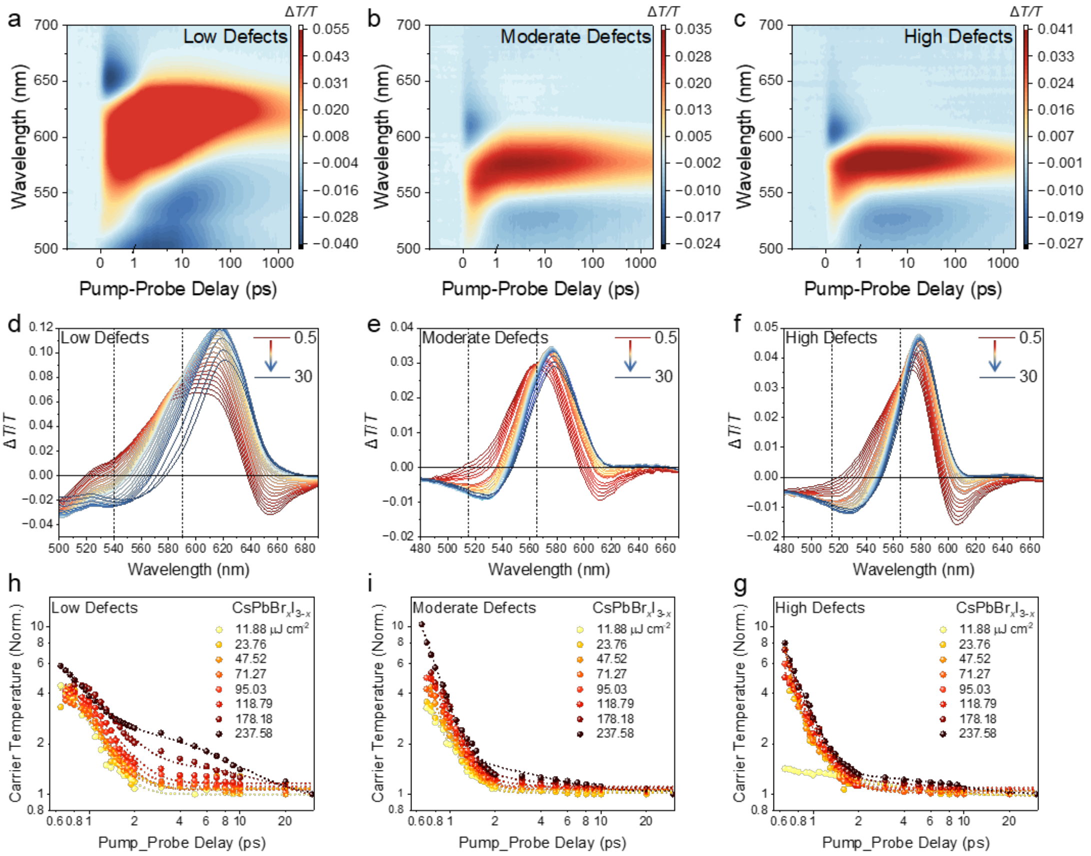

Supplementary Fig. 9 | Hot carrier cooling kinetics based on pump -probe transient absorption spectroscopy for $CsPbBr_xI_{3-x}$ NCs. a-c, TA maps, **d-f,** TA spectra and **h-g,** extracted charge-carrier temperatures *vs*. pump-probe delay of **a, d, h,** pristine (low defect density), **b, e, i,** single-purified (moderate defect density), and **c, f, g,** doubly-purified (high defect density) $CsPbBr_xI_{3-x}$ perovskite nanocrystal solutions. The maps and spectra were recorded under 237.58 μJ cm^{-2}. In **d-f**, the fitting window for obtaining hot carrier cooling kinetics is indicated with the dashed lines, and was chosen to cover the relevant energy range for hot carriers, whilst not being affected by the decay in the GSB.

Supplementary Table 3 | Fitted Cooling Kinetics for $CsPbBr_xI_{3-x}$ NCs shown in Fig. 4b. The number of decimal places quoted for the mean values and uncertainties were obtained based on numerical fitting of the carrier temperature vs pump-probe delay time. We rounded to 1 d.p. to not imply that we can measure with better than 100 fs accuracy. A biexponential model was applied to fit the decay in the carrier temperature over time. At low fluence, the overall time constants ($t_{average}$) mostly follow t_1, indicating a monoexponential decay.

Fluence μJ cm^{-2}	Carrier Density cm^{-3}	$t_{average}$ (ps)	t_1 (ps)	A_1	t_2 (ps)	A_2
Pristine (low defect density)						
11.88	2.90×10^{17}	0.5±0.1	0.5±0.1	100.0%	-	-
23.76	5.80×10^{17}	0.5±0.1	0.5±0.1	51.3%	0.6±0.1	48.7%
47.52	11.59×10^{17}	0.5±0.0	0.5±0.1	51.0%	0.6±0.0	49.0%
71.27	17.39×10^{17}	0.6±0.1	0.6±0.1	49.9%	0.6±0.1	50.1%
95.03	23.19×10^{17}	0.6±0.1	0.6±0.1	45.6%	0.6±0.1	54.4%
118.79	28.98×10^{17}	0.7±0.0	0.7±0.0	29.5%	0.7±0.1	70.5%
178.18	43.48×10^{17}	1.1±0.1	0.8±0.1	19.7%	1.2±0.0	80.3%
237.58	57.97×10^{17}	7.1±0.1	1.4±0.1	17.5%	7.3±0.1	82.5%
Singly-purified (moderate defect density)						
Fluence μJ cm^{-2}	Carrier Density cm^{-3}	$t_{average}$ (ps)	t_1 (ps)	A_1	t_2 (ps)	A_2
11.88	2.90×10^{17}	0.3±0.1	0.3±0.1	100.0%	-	-
23.76	5.80×10^{17}	0.3±0.1	0.3±0.1	99.6%	0.1±0.1	0.4%
47.52	11.59×10^{17}	0.4±0.1	0.4±0.1	93.9%	0.2±0.0	6.1%
71.27	17.39×10^{17}	0.4±0.1	0.4±0.1	68.6%	0.4±0.3	31.4%
95.03	23.19×10^{17}	0.4±0.1	0.4±0.1	65.0%	0.4±0.1	35.0%
118.79	28.98×10^{17}	0.5±0.1	0.4±0.0	64.8%	0.7±0.1	35.2%
178.18	43.48×10^{17}	0.9±0.1	0.5±0.0	60.5%	1.2±0.2	39.5%
237.58	57.97×10^{17}	4.8±0.2	0.5±0.0	58.7%	5.3±0.2	41.3%
Doubly-purified (high defect density)						
Fluence μJ cm^{-2}	Carrier Density cm^{-3}	$t_{average}$ (ps)	t_1 (ps)	A_1	t_2 (ps)	A_2
11.88	2.90×10^{17}	0.1±0.1	0.1±0.1	100.0%	-	-
23.76	5.80×10^{17}	0.1±0.0	0.1±0.0	93.5%	0.1±0.0	6.5%
47.52	11.59×10^{17}	0.2±0.1	0.1±0.1	85.9%	0.3±0.1	14.1%
71.27	17.39×10^{17}	0.3±0.1	0.1±0.1	85.4%	0.5±0.1	14.6%
95.03	23.19×10^{17}	0.3±0.1	0.2±0.2	82.2%	0.5±0.1	17.8%
118.79	28.98×10^{17}	0.4±0.2	0.2±0.1	72.9%	0.5±0.3	27.1%
178.18	43.48×10^{17}	0.9±0.0	0.2±0.0	71.6%	1.2±0.1	28.4%
237.58	57.97×10^{17}	4.5±0.0	0.2±0.0	67.8%	4.9±0.1	32.2%

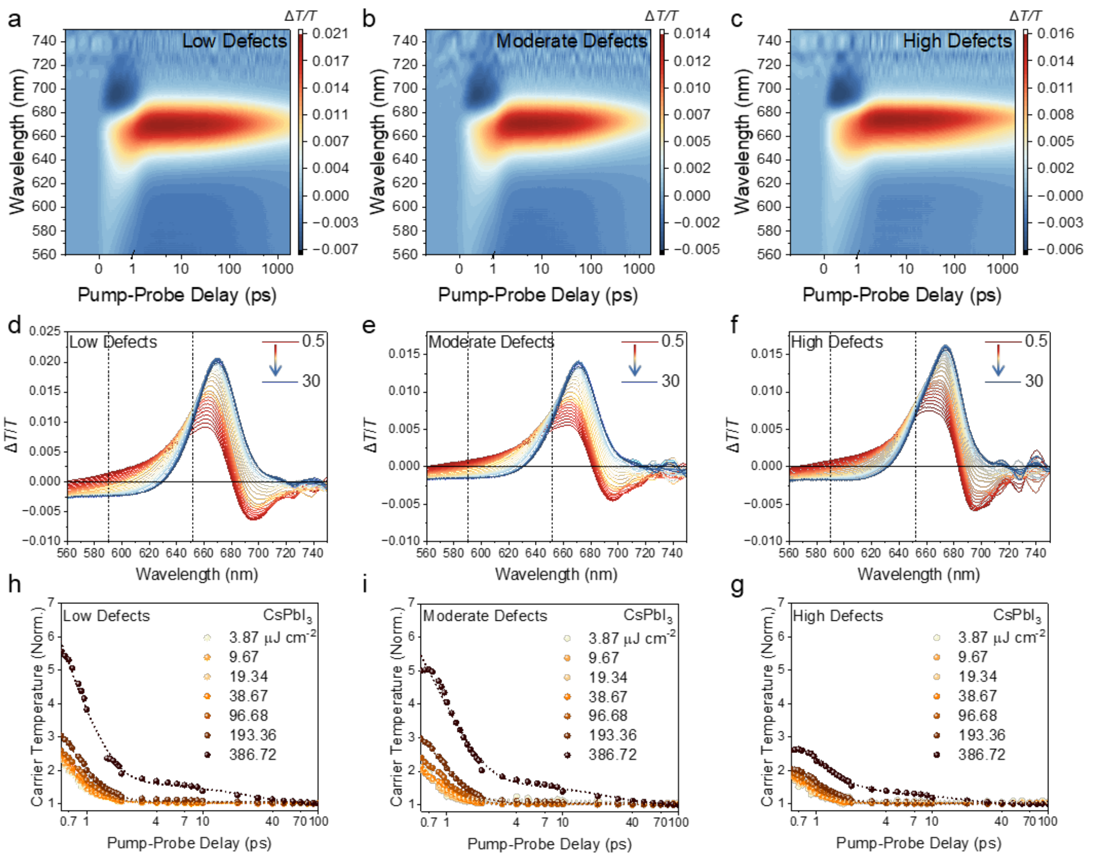

Supplementary Fig. 10 | Hot carrier cooling kinetics based on pump -probe transient absorption spectroscopy for $CsPbI_3$ NCs. a-c, TA maps, **d-f,** TA spectra and **h-g,** extracted charge-carrier temperatures *vs*. pump-probe delay of **a, d, h,** pristine (low defect density), **b, e, i,** single-purified (moderate defect density), and **c, f, g,** doubly-purified (high defect density) $CsPbBr_3$ perovskite nanocrystal solutions. The maps and spectra were recorded under 193.36 μJ cm^{-2} fluence. In **d-f**, the fitting window for obtaining hot carrier cooling kinetics is indicated with the dashed lines, and was chosen to cover the relevant energy range for hot carriers, whilst not being affected by the decay in the GSB.

Supplementary Table 4 | Fitted Cooling Kinetics for $CsPbI_3$ NCs shown in Fig. 4c. The number of decimal places quoted for the mean values and uncertainties were obtained based on numerical fitting of the carrier temperature vs pump-probe delay time. We rounded to 1 d.p. to not imply that we can measure with better than 100 fs accuracy. A biexponential model was applied to fit the decay in the carrier temperature over time. At low fluence, the overall time constants ($t_{average}$) mostly follow t_1, indicating a monoexponential decay.

Pristine (low defect density)						
Fluence μJ cm^{-2}	Carrier Density cm^{-3}	$t_{average}$ (ps)	t_1 (ps)	A_1	t_2 (ps)	A_2
3.87	0.33×10^{17}	0.3±0.1	0.3±0.1	100.0%	-	-
9.67	0.83×10^{17}	0.3±0.1	0.3±0.1	100.0%	-	-
19.34	1.66×10^{17}	0.4±0.1	0.40±0.1	100.0%	-	-
38.67	3.33×10^{17}	0.5±0.1	0.5±0.1	100.0%	-	-
96.68	8.32×10^{17}	0.6±0.2	0.6±0.2	100.0%	-	-
193.36	16.64×10^{17}	0.7±0.0	0.7±0.0	100.0%	-	-
386.72	33.29×10^{17}	13.7±1.4	0.8±0.4	66.2%	15.3±1.9	33.8%
Singly-purified (moderate defect density)						
Fluence μJ cm^{-2}	Carrier Density cm^{-3}	$t_{average}$ (ps)	t_1 (ps)	A_1	t_2 (ps)	A_2
3.87	0.33×10^{17}	0.3±0.1	0.3±0.1	100.0%	-	-
9.67	0.83×10^{17}	0.3±0.2	0.3±0.2	100.0%	-	-
19.34	1.66×10^{17}	0.4±0.3	0.4±0.3	100.0%	-	-
38.67	3.33×10^{17}	0.5±0.3	0.5±0.3	100.0%	-	-
96.68	8.32×10^{17}	0.6±0.3	0.6±0.3	100.0%	-	-
193.36	16.64×10^{17}	0.6±0.3	0.6±0.3	100.0%	-	-
386.72	33.29×10^{17}	12.1±1.6	0.8±0.1	84.2%	15.2±1.9	15.8%
Doubly-purified (high defect density)						
Fluence μJ cm^{-2}	Carrier Density cm^{-3}	$t_{average}$ (ps)	t_1 (ps)	A_1	t_2 (ps)	A_2
3.87	0.33×10^{17}	0.2±0.1	0.3±0.1	100.0%	-	-
9.67	0.83×10^{17}	0.4±0.3	0.4±0.3	100.0%	-	-
19.34	1.66×10^{17}	0.4±0.2	0.4±0.2	100.0%	-	-
38.67	3.33×10^{17}	0.4±0.2	0.4±0.2	100.0%	-	-
96.68	8.32×10^{17}	0.5±0.2	0.5±0.2	100.0%	-	-
193.36	16.64×10^{17}	0.6±0.3	0.6±0.3	100.0%	-	-
386.72	33.29×10^{17}	12.6±3.4	0.8±0.1	76.1%	14.6±3.5	23.9%

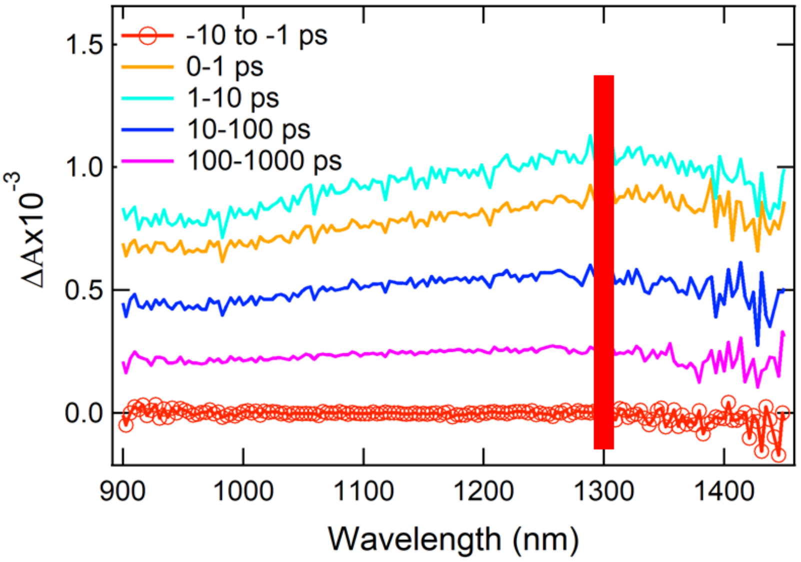

Supplementary Fig. 11 | Selection of the push energy. To select the appropriate wavelength for the push pulse, the IR transient absorption was performed in the range between 900 nm to 1450 nm wavelength. The red bar indicates that the highest absorption was reached at 1300 nm wavelength, hence its selection as the push beam wavelength for the pump-push-probe (PPP) analysis.

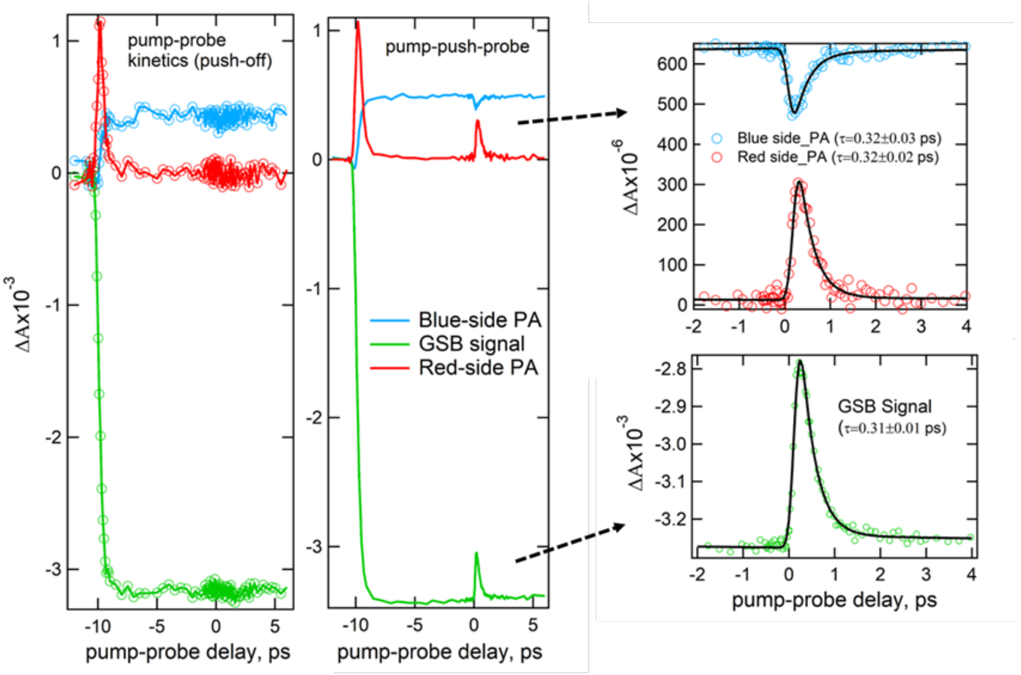

Supplementary Fig. 12 | Kinetics of GSB and PAs for the representative case of $CsPb(Br/I)_3$ NCs under PP and PPP-TA measurements. All samples are colloidal solutions, and are measured inside a 1 mm thick cuvette. The NIR push beam has an energy of 0.95 eV (1300 nm). The pump laser is a 400 nm pulse laser at 2 μJ cm^{-2}. The number of decimal places quoted for the mean values and uncertainties were obtained based on numerical fitting of the carrier temperature vs pump-probe delay time.

Supplementary Note 2: Hot Carrier Trap-Assisted Cooling Kinetic Model

The hot carrier cooling kinetic model is based on our earlier works[4, 5, 6], and modified here to include the effects of trapping. The model is described by a system of coupled differential Supplementary Eq. S6-9. The simulations of the kinetics were carried out using the Midpoint method.

$$\frac{\mathrm{d}n_{\mathrm{hot}}}{\mathrm{d}t} = \tilde{I}(t)n_{\mathrm{cold}} - \alpha n_{\mathrm{cold}} n_{\mathrm{hot}} - \phi n_{\mathrm{ph}} n_{\mathrm{hot}} - \gamma(N_{\mathrm{t}} - n_{\mathrm{t}}^{*})n_{\mathrm{hot}} \tag{S6}$$

$$\frac{\mathrm{d}n_{\mathrm{cold}}}{\mathrm{d}t} = -\tilde{I}(t)n_{\mathrm{cold}} + \alpha n_{\mathrm{cold}} n_{\mathrm{hot}} + \phi n_{\mathrm{ph}} n_{\mathrm{hot}} \tag{S7}$$

$$\frac{\mathrm{d}n_{\mathrm{ph}}}{\mathrm{d}t} = -\phi n_{\mathrm{ph}} n_{\mathrm{hot}} + \eta\left(N_{\mathrm{ph}} - n_{\mathrm{ph}}\right) \tag{S8}$$

$$\frac{\mathrm{d}n_{\mathrm{t}}^{*}}{\mathrm{d}t} = \gamma(N_{\mathrm{t}} - n_{\mathrm{t}}^{*})n_{\mathrm{hot}} \tag{S9}$$

Here, $\tilde{I}(t)$ represents the push Gaussian intensity envelope. The densities of hot and cold carriers, vacant phonons, and occupied traps are represented by n_{hot}, n_{cold}, n_{ph} and n_{t}^{*}, respectively; the coefficients for hot-cold carrier and carrier-phonon scattering are represented by α and ϕ. Upon carrier-phonon scattering, the phonon becomes occupied, before freeing with a rate constant of η. The finite total phonon density (N_{ph}) leads to the hot phonon bottleneck upon high phonon occupation. The propensity of hot carriers for trapping at defect sites is described by γ. The finite total defect density (N_{t}) leads to saturation at high excitation densities, diminishing the difference in hot carrier lifetime between low- and high-defect samples. The best overlap with experimental PPP data was achieved using the values described in Supplementary Table S5, and the initial conditions imposed were as follows:

$n_{\mathrm{hot}}(0) = 0,$

$n_{\mathrm{cold}}(0) = N_{\mathrm{pump}},$

$n_{\mathrm{ph}}(0) = N_{\mathrm{ph}},$

$n_{\mathrm{t}}^{*}(0) = 0.$

Supplementary Table 5 | Fitting parameters for numerical kinetic model describing hot carrier dynamics shown in Fig. 5d-f.

Fit parameter (unit)			$CsPbBr_3$	$CsPb(Br/I)_3$	$CsPbI_3$
Pump carrier density	N_{pump}	($\times10^{12}$ cm^{-2})	1.1	1.0	1.0
Hot-cold carrier scattering	α	($\times10^{-12}$ cm^2 ps^{-1})	1.3	1.3	1.3
Carrier-phonon scattering	ϕ	($\times10^{-12}$ cm^2 ps^{-1})	7.0	3.5	3.5
Carrier-trap scattering	γ	($\times10^{-12}$ cm^2 ps^{-1})	1.5	1.5	0.0
Phonon freeing	η	(ps^{-1})	0.15	0.15	0.03
Total phonon density	N_{ph}	($\times10^{11}$ cm^{-2})	8.0	9.5	5.5

References

1. Ye J., *et al.* Elucidating the Role of Antisolvents on the Surface Chemistry and Optoelectronic Properties of $CsPbBr_xI_{3-x}$ Perovskite Nanocrystals. *J. Am. Chem. Soc.* **144**, 12102-12115 (2022).
2. Righetto M., *et al.* Hot Carriers Perspective on the Nature of Traps in Perovskites. *Nat. Commun.* **11**, 2712 (2020).
3. Huang Y.-T., *et al.* Strong Absorption and Ultrafast Localisation in $NaBiS_2$ Nanocrystals with Slow Charge-carrier Recombination. *Nat. Commun.* **13**, 4960 (2022).
4. Carwithen B.P., *et al.* Confinement and Exciton Binding Energy Effects on Hot Carrier Cooling in Lead Halide Perovskite Nanomaterials. *ACS Nano* **17**, 6638-6648 (2023).
5. Hopper T.R., *et al.* Hot Carrier Dynamics in Perovskite Nanocrystal Solids: Role of the Cold Carriers, Nanoconfinement, and the Surface. *Nano Lett.* **20**, 2271-2278 (2020).
6. Hopper T.R., *et al.* Kinetic Modelling of Intraband Carrier Relaxation in Bulk and Nanocrystalline Lead-halide Perovskites. *Phys. Chem. Chem. Phys.* **22**, 17605-17611 (2020).
