# Supplementary figures and images for: Extending the defect tolerance of halide perovskite nanocrystals to hot carrier cooling dynamics
Source: Nat Commun. 2024 Sep 16;15:8120. doi: 10.1038/s41467-024-52377-4 (PMC11405528; doi:10.1038/s41467-024-52377-4)

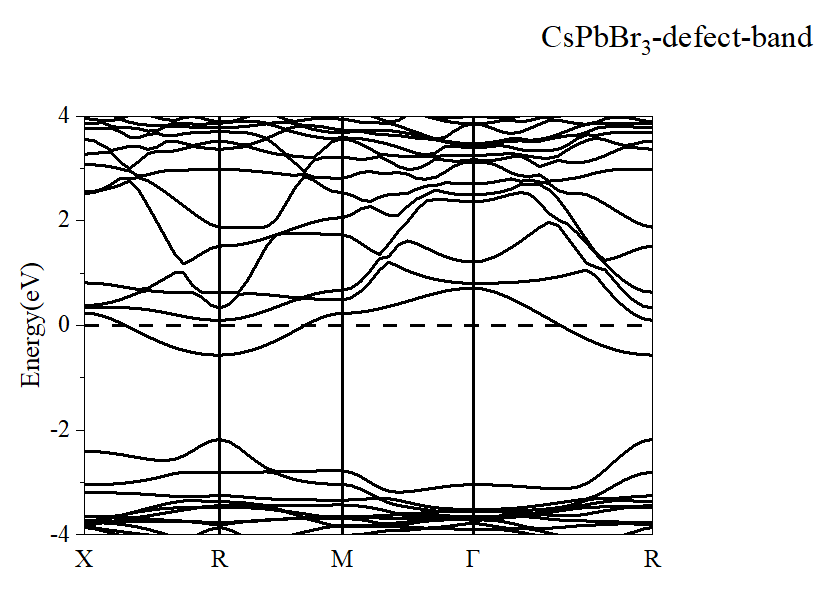

Supplement: Supplementary file 3 — Dataset 1 [file 41467_2024_52377_MOESM3_ESM.zip › Supplementary Information for DFT Calculation/CsPbBr3-defect/CsPbBr3-defect-band.png]

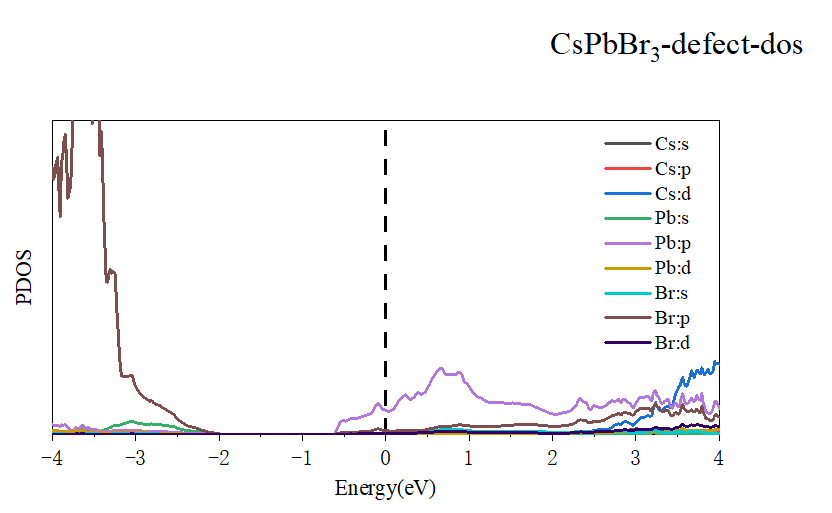

Supplement: Supplementary file 3 — Dataset 1 [file 41467_2024_52377_MOESM3_ESM.zip › Supplementary Information for DFT Calculation/CsPbBr3-defect/CsPbBr3-defect-dos.png]

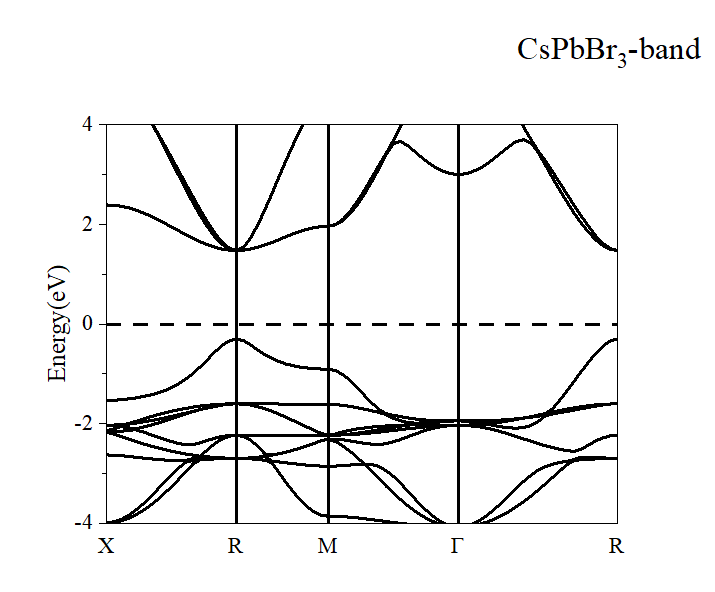

Supplement: Supplementary file 3 — Dataset 1 [file 41467_2024_52377_MOESM3_ESM.zip › Supplementary Information for DFT Calculation/CsPbBr3/CsPbBr3-band.png]

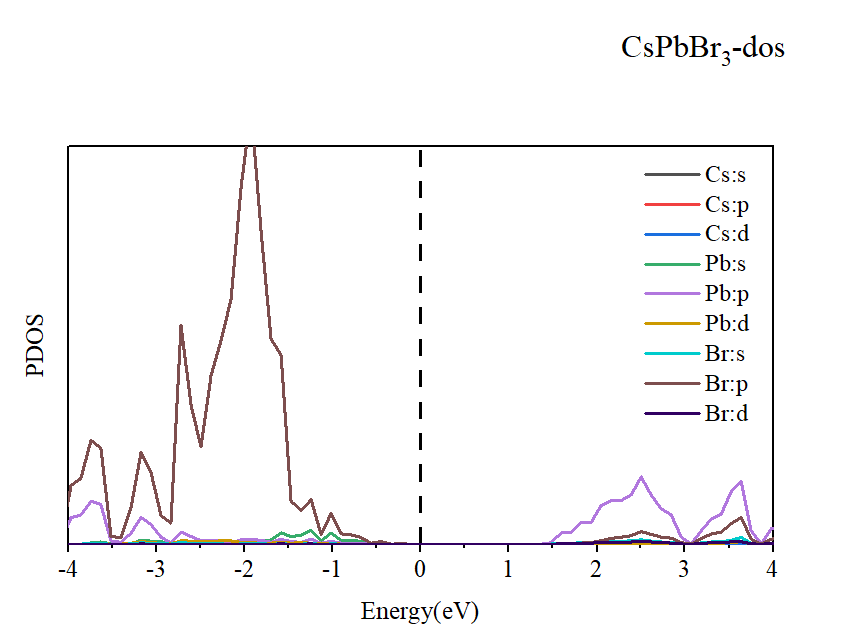

Supplement: Supplementary file 3 — Dataset 1 [file 41467_2024_52377_MOESM3_ESM.zip › Supplementary Information for DFT Calculation/CsPbBr3/CsPbBr3-dos.png]

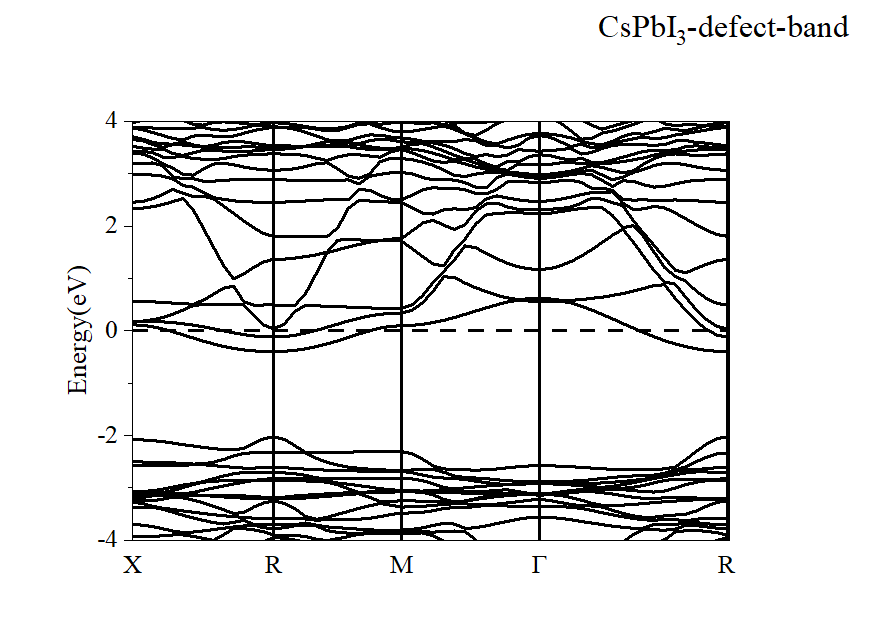

Supplement: Supplementary file 3 — Dataset 1 [file 41467_2024_52377_MOESM3_ESM.zip › Supplementary Information for DFT Calculation/CsPbI3-defect/CsPbI3-defect-band.png]

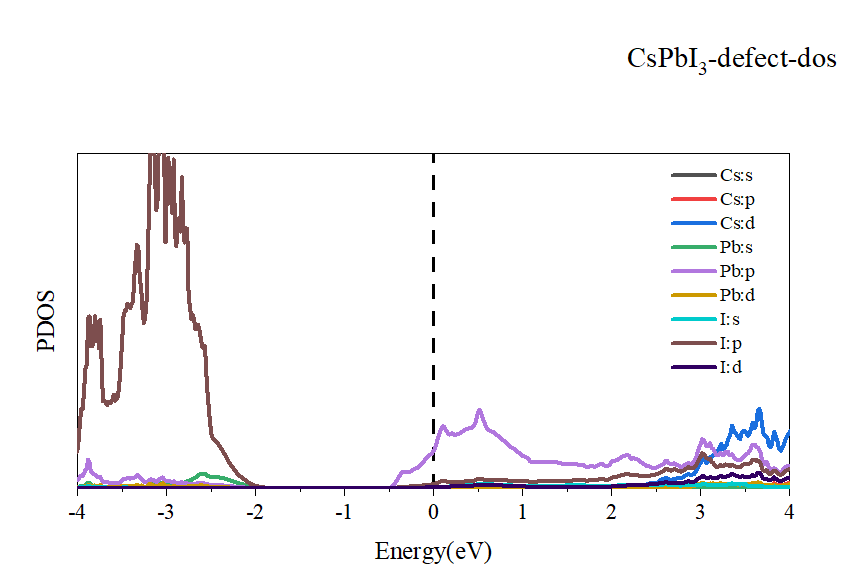

Supplement: Supplementary file 3 — Dataset 1 [file 41467_2024_52377_MOESM3_ESM.zip › Supplementary Information for DFT Calculation/CsPbI3-defect/CsPbI3-defect-dos.png]

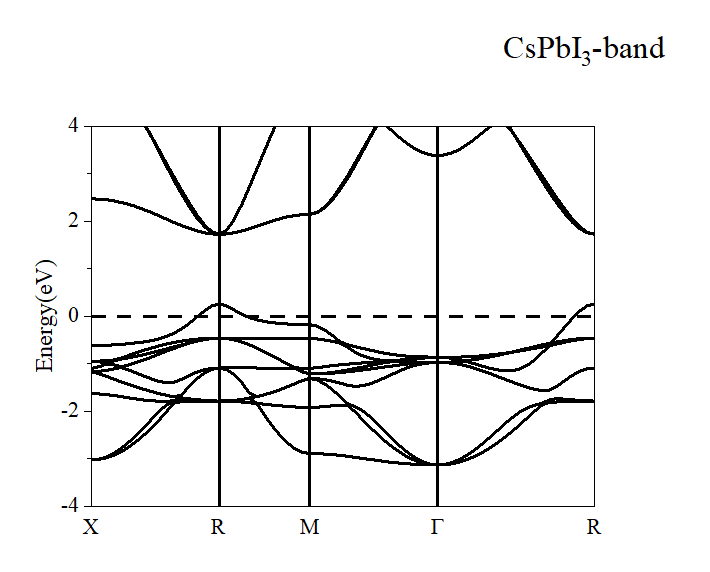

Supplement: Supplementary file 3 — Dataset 1 [file 41467_2024_52377_MOESM3_ESM.zip › Supplementary Information for DFT Calculation/CsPbI3/CsPbI3-band.png]

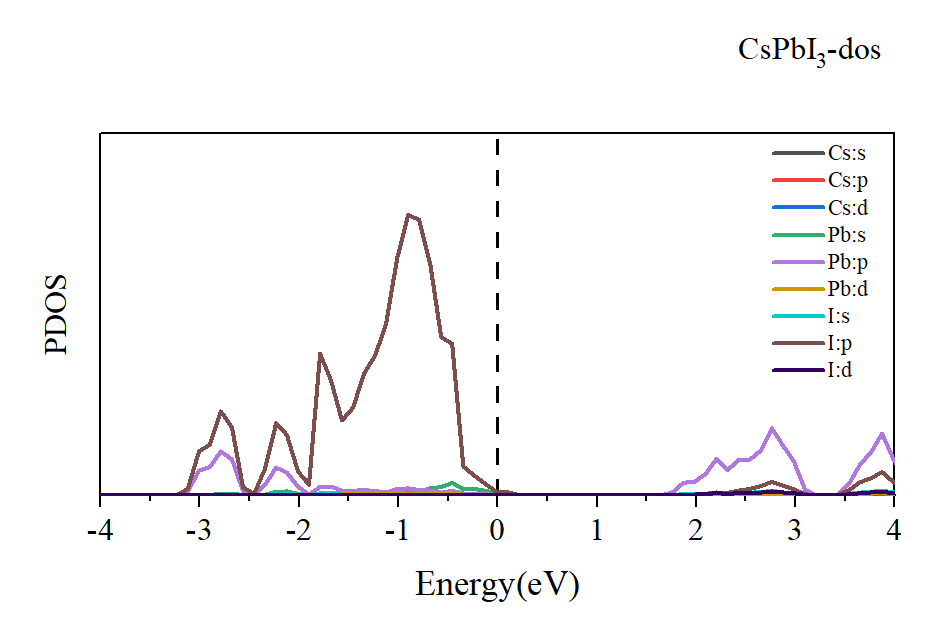

Supplement: Supplementary file 3 — Dataset 1 [file 41467_2024_52377_MOESM3_ESM.zip › Supplementary Information for DFT Calculation/CsPbI3/CsPbI3-dos.png]

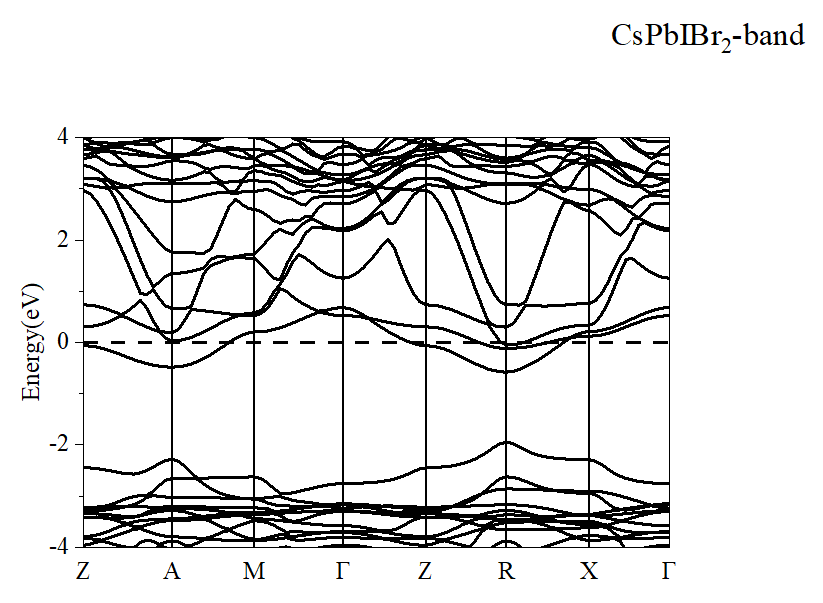

Supplement: Supplementary file 3 — Dataset 1 [file 41467_2024_52377_MOESM3_ESM.zip › Supplementary Information for DFT Calculation/CsPbIBr2-defect/CsPbIBr2-band-defect.png]

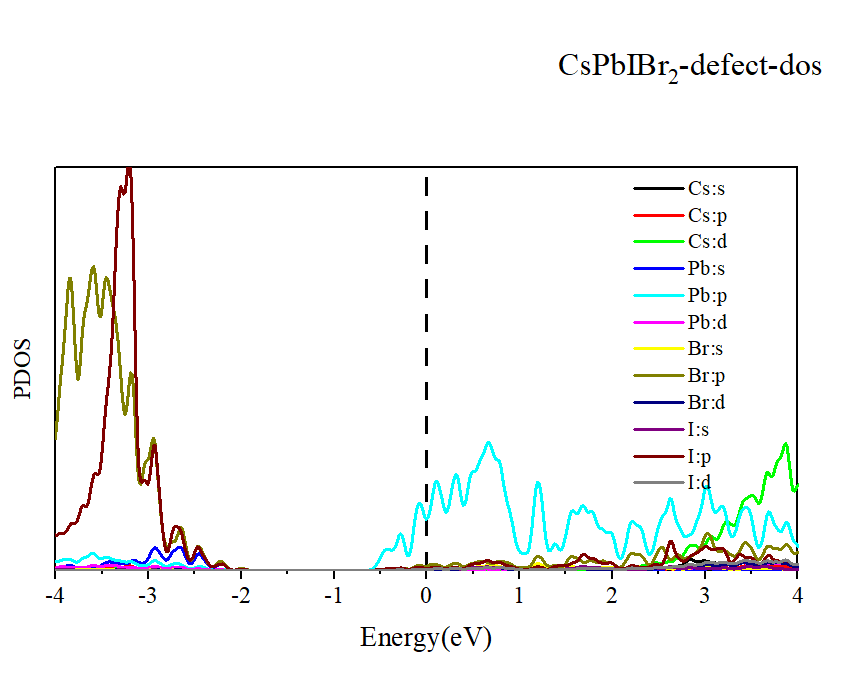

Supplement: Supplementary file 3 — Dataset 1 [file 41467_2024_52377_MOESM3_ESM.zip › Supplementary Information for DFT Calculation/CsPbIBr2-defect/CsPbIBr2-defect-dos.png]

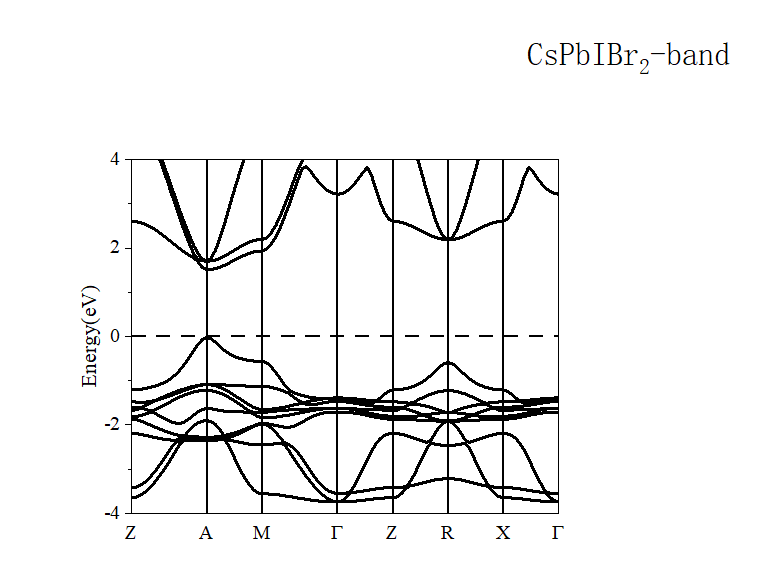

Supplement: Supplementary file 3 — Dataset 1 [file 41467_2024_52377_MOESM3_ESM.zip › Supplementary Information for DFT Calculation/CsPbIBr2/CsPbIBr2-band.png]

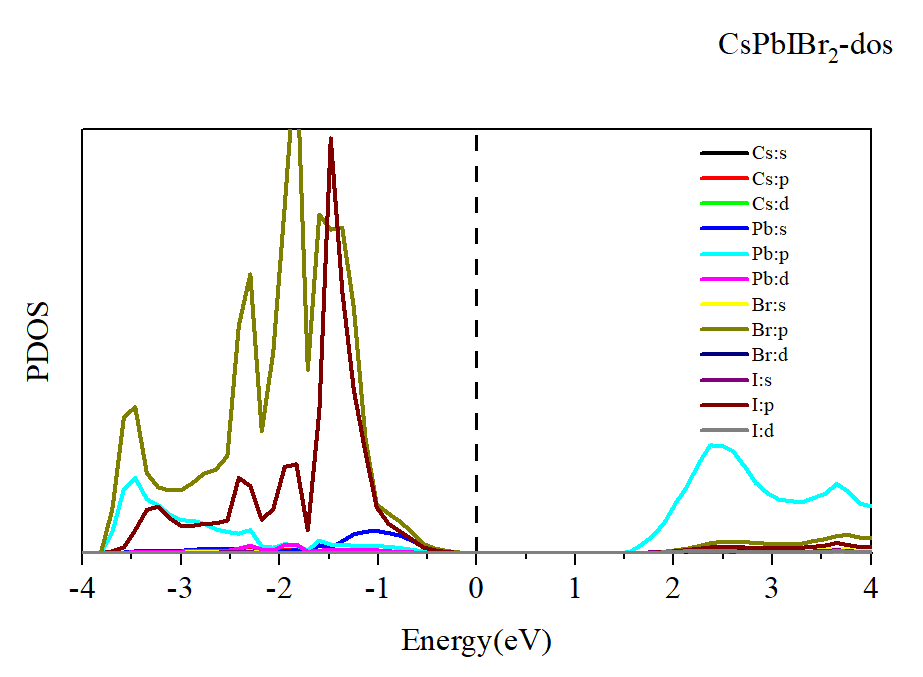

Supplement: Supplementary file 3 — Dataset 1 [file 41467_2024_52377_MOESM3_ESM.zip › Supplementary Information for DFT Calculation/CsPbIBr2/CsPbIBr2-dos.png]
